# ‘I think both of us drew strength from it’: qualitative reflections from next of kin following the death and post-mortem brain donation of a loved one with brain cancer

**DOI:** 10.1177/26323524241272106

**Published:** 2024-08-19

**Authors:** Cassandra P. Griffin, Melissa A. Carlson, Marjorie M. Walker, James Lynam, Christine L. Paul

**Affiliations:** Level 3 West, Hunter Medical Research Institute, New Lambton Heights, Newcastle, NSW 2305, Australia; School of Public Health and Medicine, The University of Newcastle, Callaghan, NSW, Australia; Hunter Medical Research Institute, Newcastle, NSW, Australia; Mark Hughes Foundation Centre for Brain Cancer Research, The University of Newcastle, Callaghan, NSW, Australia; School of Public Health and Medicine, The University of Newcastle, Callaghan, NSW, Australia; Hunter Medical Research Institute, Newcastle, NSW, Australia; School of Public Health and Medicine, The University of Newcastle, Callaghan, NSW, Australia; Hunter Medical Research Institute, Newcastle, NSW, Australia; Mark Hughes Foundation Centre for Brain Cancer Research, The University of Newcastle, Callaghan, NSW, Australia; Department of Medical Oncology, Calvary Mater Newcastle, Newcastle, NSW, Australia; School of Public Health and Medicine, The University of Newcastle, Callaghan, NSW, Australia; Hunter Medical Research Institute, Newcastle, NSW, Australia; Mark Hughes Foundation Centre for Brain Cancer Research, The University of Newcastle, Callaghan, NSW, Australia; Cancer Research, Innovation and Translation, The University of Newcastle, Callaghan, NSW, Australia

**Keywords:** brain cancer, brain donation, carer, psychosocial

## Abstract

**Background:**

Glioblastoma, a high-grade primary brain cancer, has a median survival of approximately 14 months. Post-mortem brain donation provides insight to pathogenesis along with spatial and temporal heterogeneity. Post-mortem brain biobanking programs are increasing in number and the need to understand and improve the associated human experience is pressing. This study aims to qualitatively explore the experiences of next of kin (NOK) following the death and brain donation of a loved one and to understand the impact such programs have on NOK carers.

**Method:**

We interviewed 29 NOK following the death of their loved one and subsequent brain donation. Thematic analysis was conducted on the transcribed, qualitative interviews.

**Results:**

Four themes were identified; (1) Brain donation is a straightforward decision grounded in altruism and pragmatism; (2) Supporting donors is a source of comfort, pride and empowerment; (3) Brain donation can provide meaning for suffering and tragedy and (4) Perceptions of procedures and processes when supporting a loved one to donate. Insights into areas for improvement, for example transporting donors following a home death and the role of the body bag were also noted.

**Conclusion:**

Supporting a loved one to donate their brain can be a positive experience providing a source of hope, empowerment and purpose for NOK. Data indicating areas for consideration are broadly relevant for improving the delivery of brain donation programs for future donors and their loved ones.

## Plain language summary

Understanding how loved ones feel about someone close to them donating their brain to research after their death from brain cancer

The act of donating brain tissue after death from brain cancer is a huge gift to medical research and may have an impact on the ability of the scientific community to improve outcomes for people diagnosed with brain cancers. While we understand how valuable these donations are for research, we need more work to understand how these donations impact the people who donate and those who love and support them. This paper explores the experiences of people who have lost someone to brain cancer who then went on to donate their brain tissue after their death. Through the use of interviews, it explores the impact that the donation has on a loved one or next of kin from providing a source of comfort, empowerment, pride or an alternative to ‘senseless’ suffering and tragedy. It also provides areas that should be considered by people who are facilitating brain donations to ensure that any potential, harm or upset can be minimized.

## Background

Glioblastoma is the most common primary central nervous system cancer with a median survival of 14 months despite surgical interventions, chemotherapy and radiation therapy.^1^ High-grade gliomas, including glioblastoma, have debilitating symptoms including headaches, vision loss, seizures, speech disturbance and paralysis. The impact of high-grade gliomas on the health care system is disproportionate to the incidence of disease due to high mortality and the profoundly disabling impact.^2^

It is widely understood that interventions will continue to be suboptimal until a greater understanding of brain tumour neurobiology is gained. A key facilitator in achieving such progress is access to high-quality, annotated biospecimens for translational research.^3^ While surgical specimens have traditionally been favoured for collection, recent focus has shifted towards the need for samples that afford insight into spatial and temporal heterogeneity.^4^ Post-mortem brain donation is not a new concept and substantial work has been published regarding best practice considerations for technical and logistical factors.^5–7^ However, far less is known regarding the human experience of brain biobanking, particularly in the context of brain cancer.

A systematic review published by Lin et al. aimed to conceptualise a framework for brain donation across settings (i.e. not restricted to brain cancer). Lin et al. identified four key pillars that influence decision-making.^8^ Further work published by our team (Griffin et al.^9^) aimed to characterise human experiences of brain donation further, exploring perceptions of value across brain donation settings within the existing literature. This study revealed a distinct lack of literature focusing on the experiences of people with brain cancer and their loved ones when navigating a brain donation pathway.

The expectations placed on next of kin (NOK) when caring for a person with brain cancer are substantial.^10^ Many carers report feeling simultaneously overwhelmed and helpless when their loved ones experience periods of high dependency or protracted agonal phases.^11^ Variations exist between health systems nationally and internationally; however, NOK are generally required to play a central role in providing consent for post-mortem organ donation for research or transplanation.^12,13^ NOK consent has been identified as a key factor in the ongoing shortages of donor organ tissue.^14^ Within Australia policies regarding the role of NOK differ by state; one example is the NSW legislation which requires NOK to provide consent irrespective of whether consent was provided by the donor prior to death.^15^ As such, it is essential that we gain a greater understanding of the lived experience of NOK who are supporting a loved one to participate in brain donation. This study aims to characterise experiences from NOK post-donation, recognising that responses are likely to be impacted by the lived experience of losing a loved one, ceasing carer responsibilities and completing the donation process. Such data will inform efforts to maintain the feasibility of brain donation programs for brain cancer research and minimise negative impacts on the well-being of NOK.

## Method

### Participants and setting

Participants were recruited in conjunction with the Mark Hughes Foundation (MHF) Brain Cancer Biobank Platform. Any NOK who had supported an adult with high-grade glioma^16^ to donate to the MHF Brain Cancer Biobank following their death was eligible to participate in the study. All (100%) of NOK who were approached subsequently consented to participate, leading to a sample size of 29 participants.

While we recognise that Reflexive Thematic Analysis is not as appropriately assessed by frameworks such as the Consolidated Criteria for Reporting Qualitative Research (COREQ) statement as other forms of qualitative research, the reporting of this study conforms to the COREQ statement^17^ as closely as practical. A checklist has been uploaded as Supplemental File 1.

### Procedure and measures

Potential participants were contacted by post and then followed up by telephone. The semi-structured interviews were participant-centred, allowing each participant to tell their story with interviewer clarification. The interview schedule focused on views and feelings throughout the donation process, rationale and hopes for outcomes, logistics and challenges as well as reflections on the process following the death of the donor. Interviews were conducted in-person or if necessary, via telephone. In-person interviews were conducted in the home or in a health care facility. The interviews were recorded and transcribed, including the input of any non-participants present such as spouses or adult children. The interviews were completed at 3–36 months post-donation during March 2021 to November 2023 and ranged from 19 to 60 min in duration. The interviews were conducted by the biobanking manager (CG) who is responsible for the documentation of consent with potential brain donors and responsible for liaising with NOK during the actual donation process. Sampling continued until participants provided rich breadth and depth for analysis, rather than at the point of saturation.^18^ Participants were offered the opportunity to review and correct transcripts prior to analysis however all declined this offer.

A validated ‘decisional regret scale’ (five statements with Likert-scale responses to measure post-donation remorse) was also completed by participants.^19^

### Interviewer perspective

It is acknowledged that the researcher established connections and rapport with many of the participants, in the majority of cases was present during the donation itself and is known to the participants. As such, it is recognised that the researcher is personally present in the study and contributes personal experience and insights to the interpretation of results in this phenomenological study.

### Analysis

Analysis was facilitated with the use of NVivo (Version 12 Pro, QSR International) and was conducted by two authors (CG and MC, Newcastle, Australia). All responses were coded and reflexively discussed following which a reflexive thematic analysis with narrative synthesis was conducted to explore the sequential organisation of events and understand the components of each theme within the wider brain donation paradigm. This process included individual review of data by each reviewer and a preliminary ‘coding’ phase that was then discussed between reviewers to ensure consensus regarding terminology and meaning associated with each code. These codes were used to form the basis of an initial ‘codebook’ that continued to evolve and diversify as further interviews were coded and additional codes required to represent the data. Guided by the methodology of Braun and Clarke and their six-phase process for data engagement, coding and theme development,^20^ data were coded based on key words, phrasing and expressed sentiments identified within the transcripts. While a codebook was formed, emphasis was placed on theoretical sensitivity and reflexivity. These codes were then grouped based on parallels in language and contextual factors and used to construct themes, favouring an inductive approach. This process was carried out independently by each author prior to collaborative discussion and formation of final themes. Constructed themes were illustrated and presented in narrative form with the use of demonstrative quotes to capture sentiment. It is recognised that both authors interpreted through a personal lens – one directly involved in the donation program and the other external to the program. In accordance with the *Decision Regret Scale User Manual*, we converted scores to a scale of 0–100. A score of 0 indicates no regret and 100 indicates high regret.^19^

## Results

*Participants*: All 29 participants who were invited to join the study consented to participation. Table 1 details study participation and basic demographic data along with relationship to donor. Occupational categories were informed by ISCO-08^21^ though not strictly followed to ensure heterogeneity was adequately captured.

**Table 1.** Demographics for participating NOK.

Demographic	Range/Category	*n* =
Age	20–30	1
	30–40	3
	40–50	5
	50–60	8
	60–70	9
	70–80	3
Biological Sex	Male	8
	Female	21
Occupation	Professional	14
	Clerical support	1
	Management	3
	Service worker	3
	Sales worker	1
	Technician	1
	Skilled trade	2
	Skilled agricultural	1
	Non-working adult	2
	Not documented	1
Relationship	Daughter	4
	Wife	15
	Husband	4
	Son	2
	Father	2
	Sister	1
	Mother	1

NOK, next of kin.

## Decisional regret data

Twenty-five participants completed the scale. None reported regret in relation to their donation decision, with 23 participants selecting ‘Strongly Agree’ for the positive statements intended to demonstrate support and ‘Strongly Disagree’ for the sentiments intended to demonstrate regret. One participant queried the phrasing of the statement ‘the decision was a wise one’ and opted to score this between ‘strongly agree’ and ‘agree’ which would be scored as ~12.5. Likewise, another opted for ‘agree’ and ‘disagree’ in place of ‘strongly agree’ or ‘strongly disagree’ in these instances their score for such responses would be 25/100. All others had a score of 0. In line with similar research, a score of ⩾30 is indicative of moderate to strong regret.^22^

## Thematic analysis

Four themes were identified and are described below. Broadly speaking, there did not appear to be notable differences between the responses of those who were recently bereaved and those several years post-donation. Consequently, and to preserve anonymity, the cited quotes are identified only by a non-identifying number.

### Theme 1: ‘We were just doing it, that’s it!’ – Brain donation is an uncomplicated decision grounded in altruism and pragmatism

Respondents largely dismissed the notion of an extensive decision-making ‘process’, stating that it was a ‘given’ or entirely logical ‘next step’ in their brain cancer pathway.

NOK 5 ‘It was a no-thought straight away. [Donor] is just one of those people that straight away it was – I’m donating my brain’

NOK 28 ‘It wasn’t any great decision. We’d always said that, if we could help in any way, we would give any parts of our body to help anybody. So, when [it] happened we were both “yes definitely”’.

Those who did recognise a ‘decision-making process’ expressed an overall tone of pragmatism, both in perceptions of the donor’s decision and of NOK support. Altruism and the desire to help others was at the heart of the decision-making process.

NOK 11 ‘It was a fairly easy decision. . . It really struck a chord. . ., that he wanted to do something to help people in the future. . . it was so important for him. . . He did ask what my thoughts were, and of course I just said whatever he wanted to do, I would support him’.

For many, altruism was also linked to an avoidance of waste, at times coupled with a morbid humour;

NOK 14 ‘[He would say] I’m not going to need it where I’m going, so take what you want’.

While some did discuss initial apprehension, the respect for their loved one’s autonomy and decisional capacity overrode any unease they might have held with the focus for many on the importance of respecting their loved one’s agency.

NOK 17 ‘It wasn’t really our decision, it was his decision alone, and that was one thing he wanted to do . . . it wasn’t really much of a discussion’.

NOK 25 ‘Oh I was going for and against it all night, but then it kept coming back to – well she’s thought about this for a long time. . . when she said yes, end of conversation’.

While pragmatism was prominent, the idea that sense of self is linked to brain tissue was expressed by NOK as a consideration.

NOK 12 ‘I then though, after he said he wouldn’t have his memories. . . then I thought, just had a moment to myself, where I though is that what I want to do? Am I losing a part of him?’

NOK 11 ‘His greatest asset, his greatest love – he had a very good brain. Any my thoughts were pure and simply how can we take that away? But that’s not a sensible and practical [thought]’.

### Theme 2: ‘I didn’t feel helpless. . . because I could do this last thing for him’ – Supporting donors is a source of comfort, pride and empowerment

Respondents largely framed their role in supporting their loved one’s decision as an extension of their role as carer. Taking positive actions to ensure donation proceeded empowered NOK to continue supporting their loved one, providing them a ‘mission’ or positive course of action when otherwise feelings of helplessness may have prevailed.

NOK 23 ‘His reason for the path he’s been on is to try and help others. And my reason for being on the path with him was to make sure that his donation happened’.

NOK 16 ‘And we were both very much on the same page, whatever [his] wishes were, we were going to uphold them because there’s not much else you can ever do for that person again’.

Brain donation and the sense of fulfillment it affords was reported to serve as a source of comfort for many NOK, providing hope, direction and a reason to continue during a time of immense grief – both prior to and following the donation.

NOK 11 ‘I’m just being honest. I don’t know how I would have coped if I didn’t have that goal. I think I just would’ve fallen in a heap’.

NOK 21 ‘I guess that focus, that is what got me through just one more day and one more day. And instead of having this all-consuming grief it was like a little light that I kept moving towards. . . I actually keep wondering why or how I’m still functioning. . . and I know it’s because she got her wish . . . . when faced with no choices, she still got one at the end. And I have to take something from that’.

NOK 25 ‘And we’d talk about how, even though you’re finding the small, little light in all this crap, without that, where would we be? Where would we be without her donating her brain? And Dad would say, “I’d have gone bush”’.

An intense representation of this was described by a donor’s daughter who recalled a time where she was consciously willing the increased growth of the tumour, wanting the best possible research sample to be collected.

NOK 1 ‘I did have a thought at that point, I wanted it to grow as much as it could to get as much as possible. I think that’s a weird way of viewing things, it was never going to be cured at that point so I just wanted as much tumour as possible to get the research possibilities. . . it turned something that was very, very tragic into there might be purpose where some good may come from it’.

Many NOK expressed a desire to publicly celebrate brain donation as a legacy following the donor’s death. For one NOK, while the concept of legacy did not resonate when specifically asked, she later described brain donation pragmatically as something to be included in his funeral notice along with all of his achievements. Likewise, a mother repeatedly lamented omitting her son’s donation from his eulogy and was committed to sharing this with the community through other means.

NOK 19 ‘It’s important that people know [about his donation]. I also put all his academic achievements in there. I thought that putting that notice in the [newspaper] summarised all his achievements. . . I put it in there because I thought it was important’.

NOK 27 ‘I still punish myself to this day that I forgot to put [his donation] in the Eulogy. Because there are and were so many people there. . . I think that’s a big thing that people need to know. . . I kick myself because I was proud that he made that decision’.

### Theme 3: ‘His death has had some sort of purpose’ – Brain donation can provide meaning for suffering and tragedy

Many participants described a sense of purpose resulting from brain donation, suggesting that meaning for their loved one’s suffering was afforded to them by the act.

NOK 1 ‘It’s not all for nothing. There’s the possibility of something more coming from a tragic event’.

NOK 15 ‘I feel that he’s made some sort of difference. His death has had some sort of purpose’.

The importance of donation providing a sense of meaning was also mentioned when participants were asked to consider how they may have felt had the donation not proceeded or had they decided against it.

NOK 1 ‘To not do it? I think it would have been more distressing, that there was no reason for it. . . . It would have just been burned with her, and there’s no possibility of anything coming from it’.

There is a prevailing tone of gratitude within the data, one NOK expressing that not only did the donation provide meaning but afforded a sense of grace and dignity both to themselves and the donor.

NOK 21 ‘I hope that others get a chance, if they’re losing a loved one, I hope that they get the chance to lose someone with this grace. The dignity that it gave her and me I can’t ever thank anyone for enough, I can’t’.

This gratitude extends to discussions of legacy. References to legacy were varied; however, phrases such as ‘living on’ and ‘still fighting’ were used to express that many NOK feel their loved one endures and is still present in their legacy.

NOK 11 ‘knowing that after his passing he could still contribute in some way’.

NOK 20 ‘I feel more. . . at peace myself. . . knowing she got everything she wanted and knowing that there’s still a piece of her still doing, still helping. I know she’s up there just trying to help other people’.

NOK 21 ‘It definitely gave me something concrete to care about and to feel good about. I’m losing my sister, however I did all I could do so that she would live on in some way’.

The data also indicated that the physical location of the biobank was linked to the legacy of the donor with NOK spending time in the vicinity of the building to remain close to their loved ones.

NOK 1 ‘I have her ashes at home, but there’s still a part of her elsewhere. . . I feel like she’s part of that building. . . when I go in there, it’s a warm fuzzy feeling that she’s there’.

NOK 27 ‘I still, when I walk up there, because I walk up to the building virtually every day, and I always sort of have a look at it. . . I find it very comforting to know that he might still be up there’.

### Theme 4: ‘I can still remember the zipping up of the bag’ – Perceptions of procedures and processes when supporting a loved one to donate

Respondents expressed a number of concerns including anxiety over meeting timeframes once the donor had passed, paperwork issues and a general concern about relying on hospital staff. In addition to these logistical concerns, many cited the impact the body bag had on exacerbating grief or causing distress. In some cases, the body bag was a symbol of finality and of their loved one being truly gone.

NOK 1 ‘They have to take them. So it wasn’t the trauma of her being taken to donate her brain. It was the trauma of this is it. They’ve zipped up the bag. That’s it, she’s gone’.

NOK 10 ‘We were all waiting for the funeral people to come and get him. And then putting him in the bag, ready to come here – that sort of hit me’.

NOK 2 ‘They were really nice guys, but they were also. . . seeing a black body bag with your parent? Other than that, that was it’.

For others, the physical act of removing their loved one created a sense memory that ‘haunted’ the individual and provided an ongoing source of upset.

NOK 24 ‘And they put her body in a bag, so I just closed my eyes and couldn’t really look at it. . . I can imagine it, I can hear the wheels, things like that’.

Despite the prevailing negativity associated with the body bag, NOK recognise that the distress that can be caused by the appearance and use of the body bag is likely to occur irrespective of brain donation.

NOK 1 ‘It’s the zipping up of the bag. And I think that would have happened anyway regardless of the donation’.

However, for one NOK, their response was contrary to the above – indicating that the body bag provided them with a means by which to distance themselves from the grief and from their personal connection with the donor.

NOK 17 ‘It seems like, I think, the moment of clarity is when they zipped up the body bag. . . as soon as the bag was zipped up, it was like delivering a package, like helping load up a truck. . . it took the emotion away from it’.

This is also supported by the idea that the act of brain donation and of the donor physically ‘leaving’ the NOK for an agreed purpose, rather than the NOK choosing to mark the end of their carer role and physically leave the donor, may provide a source of comfort.

NOK 24 ‘I honestly don’t know what that experience would’ve been like if, instead, I had to make that choice to walk away. I would imagine that, perhaps, I would have the same sort of feeling of not being ready to let go, whereas that was somewhat made a little bit easier by the fact that she did have somewhere to go’.

NOK 21 ‘For me, it wasn’t the end. It was the next step in this journey’.

This can be contrasted to the experience of a NOK who, while providing clear ongoing consent for donation, did not want to engage or discuss the process at the time of death and described her experience of leaving her husband at the hospice and ending her carer role. She later expressed that she wished that she had stayed and waited for him to go.

NOK 29 ‘I don’t know whether I did the right thing of going before he went. . . it was just such an empty, lonely thing going away from the hospice that day’.

Overall, the data indicated that while elements of the process, such as the body bag, could be perceived as confronting, NOK found that supporting a loved one to donate their brain was ‘not difficult’ or challenging.

NOK 26 ‘Yes. Oh, yes, I never felt alone in that. The staff were always reassuring me that I didn’t have to worry about it, they had it all under control. I was happy. If you could say happy, that’s a bad word, isn’t it? But I was, yes, the process was easy for me. Everything was easy for me, I didn’t have to worry about anything’.

NOK 5 ‘There was no hard part, not for us’.

NOK 27 ‘I wouldn’t have said there was anything hard about it for me. . . it was carried out very well, very professionally. It just went so smoothly, and I wouldn’t have said there was anything hard about it at all’.

## Discussion

### Decisional regret

Overwhelmingly, the data indicated that NOK did not regret supporting a loved one to donate their brain. The decisional regret data support the qualitative data illustrating the sense of pride NOK felt at honouring their loved ones’ wishes. This is supported by the work of Millar et al.^23^ who found that NOK contacted following the death of a loved one due to suicide did not experience further distress when approached to donate and that NOK who did agree to donate did not later regret their decision. Furthermore, the Millar et al. study indicated that the vast majority of families participating in the study felt all bereaved families should be afforded the opportunity to participate in brain banking.^23^

These findings are analogous to those well documented in the transplantation literature – that organ donation can afford NOK a sense of comfort and pride during a period of immense grief.^24,25^ In their work characterising the role of nursing staff in organ transplantation Riley and Coolican identified that the ability of NOK to participate in an end-of-life decision affords an opportunity to overcome feelings of helplessness and powerlessness while also generating a sense of meaning in senseless tragedy.^26^ These findings reflect those of Eatough et al. who identified that enabling a loved one to play a critical role and ensure donation proceeded was a welcome distraction for NOK, describing it as a ‘last act’ that provided a sense of focus, control, relief and comfort.^27^

Furthermore, Eatough found that the control afforded by participating in brain donation eased the strain of relinquishing care after protracted illness, proving a much-needed practical focus. This supports the findings within Theme 2, which described the role brain donation can play by offering empowerment or presenting an ‘antidote’ to helplessness for carers. This finding suggested that engagement with brain donation protocols may serve as a positive psychosocial intervention for NOK when appropriate and aligned with the wishes of the donor.

### Legacy and brain exceptionalism

The notion of ‘legacy’ is recurrent within the data and can be explored further in terms of sub-themes and contextual factors. ‘Legacy’ can be synonymous with pride and can be linked to minor themes of ‘awareness’ through desires to share and celebrate the act of donation. This is supported by the work of Vale-Taylor who explored remembrance activities for the bereaved and identified the importance of establishing ritual or remembrance within the community following the death of a loved one.^28^ This suggests that brain donation provides a positive focal point for remembrance, assisting in fulfilling this psychosocial need for bereaved NOK.

Brain exceptionalism – the idea that the brain holds special significance or a unique relationship to the soul – was evident in the study data concerning loss of memories and loss of self. Within the literature, brain exceptionalism has both positive and negative implications for brain donation, many of which are relative to cultural context. In their work investigating the views towards brain donation of Taiwanese patients, Wu et al. discuss various views held by the community, identifying a belief that the soul may not leave the body for up to 8 h and thereby undermining the cultural safety of rapid autopsy.^29^ Alternatively, a participant in Eatough et al.’s study articulated a belief that an individual would ‘live on’ and ‘travel the world’ due to the ongoing ‘work’ of his brain tissue.^27^ The latter which carries a distinctly ‘hopeful’ and positive tone is particularly relevant to this study and was demonstrated by NOK expressing connections with the building and the ongoing ‘work’ of their loved one – insinuating that ‘self’ endures along with the remaining tissue. These insights suggest that brain donation can provide a source of comfort in grief, providing an ongoing means to remain connected to the deceased. This is supported by the transplantation literature which outlines a growing trend in bereaved NOK expressing desire to connect with the recipients of organ transplantations.^30^

In the work of Bolt et al. it was found that relatives often struggle with persistent feelings of ‘restlessness’ following a post-mortem organ donation and that many of them later developed a level of personal attachment to donated organs.^30^ The study explored further the additional relevance for parents of deceased donors with respect to thoughts of ‘living on’ through donation. This included several references to a desire to nurture or protect the organ or indicating possessiveness and ownership over the donated organ. These insights on the multi-faceted nature of legacy and the impact it can have on the grieving process for NOK suggest that discussions of legacy, including the discussions of the physical biobank structure where the tissue will be stored, would be a valuable consideration during the consent process for brain donation to ensure maximal psychosocial benefit for NOK.

### Areas for consideration and improvement

The data indicated a number of key areas that could be reviewed to reduce the potential for emotional harm during the brain donation process. The most prominent of these was the need for further consideration on the process of removal and transport of the deceased, particularly in the case of a home death. While not exclusively viewed as negative, the body bag for many was a cause of distress and an instigator of acute grief. Additional education as to how this process will take place, with specific attention to the physical removal of the deceased and the practical processes, that is, use of a trolley, body bag, removal of medical equipment, etc., may assist in reducing the potential for distress in these situations. In the case of home deaths, it may be possible for additional educational support to be provided by home care providers or palliative care/end-of-life care providers equipped to discuss the physical realities of death and patient management.

Furthermore, the data indicates that NOK feel the action of a loved one being physically removed or ‘taken’ for donation was a comfort or preferred alternative to ‘walking away’ or leaving the deceased after an extended period of care. This is supported by the work of Fridh et al. who describes the experience of arriving to the intensive care ward with a living human being and walking out with ‘a bag of clothes’.^31^ In this context, the notion expressed in our data that brain donation allows the ‘journey to continue’ and the deceased to move to the next phase may explain the reflection from NOK in this cohort that having the donor ‘taken’ was easier than walking away. If this is indeed true then further research into end-of-life care, specifically the point of separation for the deceased and their carer, could enable the development of robust guidelines and educational materials to minimise distress for carers both in a brain donation context and in broader settings.

## Limitations

It is noted that the ratio of male to female participants is 8:21 within this study; however, it is understood that brain cancer has a higher incidence in men than in women.^32^ While this sample distribution is somewhat reflective of the nature of disease, it is insufficient to explain the lack of gender parity and serves as a limitation of the study. Further investigation into biological sex and carer roles may provide additional insight to explain the disparity.

## Conclusion

This study indicates that the experiences of NOK supporting a loved one to donate their brain have a beneficial impact on experiences of grief – both anticipatory and realised. By providing a source of hope, empowerment and purpose, brain donation can serve as an antidote to helplessness and a gateway to establishing an enduring legacy of immense comfort to bereaved NOK. There are a number of key areas for further review and additional characterisation of logistical processes may assist in improving brain donation programs, maximising value and minimising harms. This study addressed a current gap in knowledge with respect to the experiences of those caring for an adult loved one donating their brain following a brain cancer diagnosis, recognising some of the unique challenges and opportunities relevant to this cohort and works to ensure that as similar programs increase in number, so too does the scope of positive psychosocial impact for NOK.

## Supplemental Material

sj-pdf-1-pcr-10.1177_26323524241272106 – Supplemental material for ‘I think both of us drew strength from it’: qualitative reflections from next of kin following the death and post-mortem brain donation of a loved one with brain cancer Supplemental material, sj-pdf-1-pcr-10.1177_26323524241272106 for ‘I think both of us drew strength from it’: qualitative reflections from next of kin following the death and post-mortem brain donation of a loved one with brain cancer by Cassandra P. Griffin, Melissa A. Carlson, Marjorie M. Walker, James Lynam and Christine L. Paul in Palliative Care and Social Practice

## Declarations

**Ethics approval and consent to participate:** Ethics approval was granted for this study by the Hunter New England Human Research Ethics Committee (HNEHREC 2018/ETH00261). Full, informed consent was obtained from all participants.

**Consent for publication:** Written consent for publication was gained prior to the interviews commencing.

**Availability of data and materials:** The datasets generated and/or analysed during the current study are available from the corresponding author on reasonable request.

## References

[1] Tomoszková S Škarda J Lipina R. Potential diagnostic and clinical significance of selected genetic alterations in glioblastoma. Int J Mol Sci 2024; 25(8):. 10.3390/ijms25084438 PMC11050250 38674026

[2] Patel AP Fisher JL Nichols E , et al. Global, regional, and national burden of brain and other CNS cancer, 1990–2016: a systematic analysis for the Global Burden of Disease Study 2016. Lancet Neurol 2019; 18(4): 376–393. 30797715 10.1016/S1474-4422(18)30468-X PMC6416167

[3] Aldape K Brindle KM Chesler L , et al. Challenges to curing primary brain tumours. Nat Rev Clin Oncol 2019; 16(8): 509–520. 30733593 10.1038/s41571-019-0177-5 PMC6650350

[4] Griffin CP Paul CL Alexander KL , et al. Postmortem brain donations vs premortem surgical resections for glioblastoma research: viewing the matter as a whole. Neurooncol Adv 2022; 4(1): vdab168. 10.1093/noajnl/vdab168 PMC8760897 35047819

[5] White K Yang P Li L , et al. Effect of postmortem interval and years in storage on RNA quality of tissue at a repository of the NIH NeuroBioBank. Biopreserv Biobank 2018; 16(2): 148–157. 29498539 10.1089/bio.2017.0099 PMC5906728

[6] Carlos AF Poloni TE Medici V , et al. From brain collections to modern brain banks: a historical perspective. Alzheimers Dement 2019; 5: 52–60. 10.1016/j.trci.2018.12.002 PMC6365388 30775417

[7] Vonsattel JPG Del Amaya MP Keller CE . Twenty-first century brain banking. Processing brains for research: the Columbia University methods. Acta Neuropathol 2008; 115: 509–532. 17985145 10.1007/s00401-007-0311-9 PMC2292479

[8] Lin M-JP Jowsey T Curtis MA . Why people donate their brain to science: a systematic review. Cell Tissue Bank 2019; 20(4): 447–466. 31538265 10.1007/s10561-019-09786-3 PMC6863784

[9] Griffin CP Bowen JR Walker MM , et al. Understanding the value of brain donation for research to donors, next-of-kin and clinicians: a systematic review. PLoS One 2023; 18(12): e0295438. 10.1371/journal.pone.0295438 PMC10732432 38117774

[10] Aoun SM Deas K Howting D , et al. Exploring the support needs of family caregivers of patients with brain cancer using the CSNAT: a comparative study with other cancer groups. PLoS One 2015; 10(12): e0145106. 10.1371/journal.pone.0145106 PMC4682982 26679505

[11] Flechl B Ackerl M Sax C , et al. The caregivers’ perspective on the end-of-life phase of glioblastoma patients. J Neurooncol 2013; 112(3): 403–411. 23412776 10.1007/s11060-013-1069-7

[12] Rodrigue JR Cornell DL Howard RJ. The instability of organ donation decisions by next-of-kin and factors that predict it. Am J Transplant 2008; 8(12): 2661–2667. 18853951 10.1111/j.1600-6143.2008.02429.x PMC2588486

[13] Rosenblum AM Horvat LD Siminoff LA , et al. The authority of next-of-kin in explicit and presumed consent systems for deceased organ donation: an analysis of 54 nations. Nephrol Dial Transplant 2012; 27(6): 2533–2546. 22121233 10.1093/ndt/gfr619 PMC3363979

[14] Holthe E Husby VS . Barriers to organ donation: a qualitative study of intensive care nurses’ experiences. Dimens Crit Care Nurs 2023; 42(5): 277–285. 37523727 10.1097/DCC.0000000000000596

[15] Governemnt N. Non-coronial post mortem. In: Health N , editor. 2013.

[16] Mahajan S Suri V Sahu S , et al. World Health Organization classification of tumors of the central nervous system 5th edition (WHO CNS5): what’s new? Indian J Pathol Microbiol 2022; 65(Suppl 1): S5–S13. 10.4103/ijpm.ijpm_48_22 35562129

[17] Tong A Sainsbury P Craig J. Consolidated criteria for reporting qualitative research (COREQ): a 32-item checklist for interviews and focus groups. Int J Qual Health Care 2007; 19(6): 349–357. 17872937 10.1093/intqhc/mzm042

[18] Braun V Clarke V. One size fits all? What counts as quality practice in (reflexive) thematic analysis? Qual Res Psychol 2021; 18(3): 328–352.

[19] O’Connor A. User Manual – Decision Regret Scale. Ottawa, Canada: Ottawa Hospital Research Institute, 1996.

[20] Braun V Clarke V Hayfield N. Thematic analysis: a reflexive approach. In: The sage handbook of qualitiative research in psychology. London, UK: Sage Publications, 2023.

[21] Tijdens KG . The importance of occupation coding quality: lessons for EU-SILC from SHARE and other international surveys. In: Lyberg L Lynn P (eds.) Improving the measurement of poverty and social exclusion in Europe: reducing non-sampling errors Luxembourg: Publications Office of the European Union, (pp. 413–426).

[22] Robertson EG Wakefield CE Tsoli M , et al. Parents’ experiences of postmortem tumor donation for high-grade gliomas: benefits and suggested improvements. Neurooncol Adv 2021; 3(1): vdab087. 10.1093/noajnl/vdab087 PMC8386242 34458732

[23] Millar T Walker R Arango JC , et al. Tissue and organ donation for research in forensic pathology: the MRC Sudden Death Brain and Tissue Bank. J Pathol 2007; 213(4): 369–375. 17990279 10.1002/path.2247

[24] Ormrod JA Ryder T Chadwick RJ , et al. Experiences of families when a relative is diagnosed brain stem dead: understanding of death, observation of brain stem death testing and attitudes to organ donation. Anaesthesia 2005; 60(10): 1002–1008. 16179046 10.1111/j.1365-2044.2005.04297.x

[25] Flodén A Kelvered M Frid I , et al. (eds.) Causes why organ donation was not carried out despite the deceased being positive to donation. Transplant Proc 2006; 38: 2619–2621. 17098016 10.1016/j.transproceed.2006.07.031

[26] Riley LP Coolican MB. Needs of families of organ donors: facing death and life. Crit Care Nurse 1999; 19(2): 53. 10401302

[27] Eatough V Shaw K Lees A. Banking on brains: insights of brain donor relatives and friends from an experiential perspective. Psychol Health 2012; 27(11): 1271–1290. 22452631 10.1080/08870446.2012.669480

[28] Vale-Taylor P. ‘We will remember them’: a mixed-method study to explore which post-funeral remembrance activities are most significant and important to bereaved people living with loss, and why those particular activities are chosen. Palliat Med 2009; 23(6): 537–544. 19304810 10.1177/0269216309103803

[29] Wu KC-C Fukushi T . Neuroethics in Taiwan: could there be a Confucian solution? East Asian Sci Technol Soc 2012; 6(3): 321–334.

[30] Bolt SH Witjes M van den Ende B. Restless feelings: desiring direct contact after postmortem organ donation. Omega 2020; 82(1): 42–62. 30217124 10.1177/0030222818800207

[31] Fridh I Forsberg A Bergbom I. Close relatives’ experiences of caring and of the physical environment when a loved one dies in an ICU. Intensive Crit Care Nurs 2009; 25(3): 111–119. 19112022 10.1016/j.iccn.2008.11.002

[32] AIHW. Brain Cancer in Australia Statistics: Australian Government: Cancer Australia, https://www.canceraustralia.gov.au/cancer-types/brain-cancer/statistics (2023, Last accessed February 2024).

